# Internet-delivered cognitive therapy for PTSD: a development pilot series

**DOI:** 10.3402/ejpt.v7.31019

**Published:** 2016-11-08

**Authors:** Jennifer Wild, Emma Warnock-Parkes, Nick Grey, Richard Stott, Milan Wiedemann, Lauren Canvin, Harriet Rankin, Emma Shepherd, Ava Forkert, David M. Clark, Anke Ehlers

**Affiliations:** 1 Department of Experimental Psychology, University of Oxford, Oxford, UK; 2 Oxford NIHR Cognitive Health Clinical Research Facility, Oxford, UK; 3 NIHR Mental Health Biomedical Research Centre at South London and Maudsley NHS Foundation Trust and King's College London, London, UK

**Keywords:** PTSD, posttraumatic stress disorder, cognitive therapy, Internet-based treatment, trauma

## Abstract

**Background:**

Randomised controlled trials have established that face-to-face cognitive therapy for posttraumatic stress disorder (CT-PTSD) based on Ehlers and Clark's cognitive model of PTSD is highly effective and feasible with low rates of dropout. Access to evidence-based psychological treatments for PTSD is insufficient. Several studies have shown that therapist-assisted treatment delivery over the Internet is a promising way of improving access to cognitive behavioural therapy interventions.

**Objective:**

To develop an Internet version of CT-PTSD that significantly reduces therapist contact time without compromising treatment integrity or retention rates.

**Methods:**

We describe the development of an Internet version of CT-PTSD. It implements all the key procedures of face-to-face CT-PTSD, including techniques that focus on the trauma memory, such as memory updating, stimulus discrimination and revisiting the trauma site, as well as restructuring individually relevant appraisals relating to overgeneralisation of danger, guilt, shame or anger, behavioural experiments and planning activities to reclaim quality of life. A cohort of 10 patients meeting DSM-IV criteria for PTSD worked through the programme, with remote guidance from a therapist, and they were assessed at pre- and post-treatment on PTSD outcome, mood, work and social adjustment and process measures.

**Results:**

No patients dropped out. Therapists facilitated the treatment with 192 min of contact time per patient, plus 57 min for reviewing the patient's progress and messages. Internet-delivered CT-PTSD was associated with very large improvements on all outcome and process measures, with 80% of patients achieving clinically significant change and remission from PTSD.

**Conclusions:**

Internet-delivered cognitive therapy for PTSD (iCT-PTSD) appears to be an acceptable and efficacious treatment. Therapist time was reduced to less than 25% of time in face-to-face CT-PTSD. Randomised controlled trials are required to evaluate systematically the acceptability and efficacy of iCT-PTSD.

**Highlights of the article:**

- iCT-PTSD was acceptable to patients and associated with very large improvements on PTSD outcome and process measures.
- iCT-PTSD required substantially less therapist time than CT-PTSD.
- iCT-PTSD may be associated with changes in employment status.
- Randomised controlled trials are required to systematically evaluate the acceptability and efficacy of iCT-PTSD.

Posttraumatic stress disorder (PTSD) is a common problem that develops in some people after trauma and is linked to high rates of comorbidity, chronic disability and long-term healthcare costs (Kessler, 2000). Trauma-focused cognitive behavioural therapy (TF-CBT) has an established evidence base, and numerous guidelines recommend this approach as a first-line treatment for the disorder (e.g., American Psychiatric Association [APA], 2004; Australian Centre for Posttraumatic Mental Health, 2013; Foa, Keane, Friedman, & Cohen, 2005; National Institute of Health and Clinical Excellence [NICE], 2005; Schnyder et al., 2015; Stein et al., 2009; Veterans Health Administration and Department of Defense, 2004). However, many patients with PTSD are unable to access treatment because resources are limited, they may fear stigmatisation and embarrassment (Hoge et al., 2004, 2014), hold negative beliefs about mental health services (Blais, Tsai, Southwick, & Pietrzak, 2015) or live in remote areas not well served by treatment centres. If left untreated, PTSD can be complicated by other disorders, such as depression and substance misuse (Kessler, Sonnega, Bromet, Hughes, & Nelson, 1995). Clearly, there is a need for effective and efficient treatments, which have greater opportunity to reach those in need. Internet-based trauma-focused treatments for PTSD may be best placed to reach this need. They offer greater anonymity than face-to-face treatments (e.g., Lange, Van De Ven, Schrieken, & Emmelkamp, 2001), are easily accessible, can be delivered at a time that suits the client and require less therapist time than face-to-face treatments (e.g., Klein et al., 2010).

In their meta-analysis, Kuester, Niemeyer, and Knaevelsrud (2016) found that current Internet-based treatments for PTSD were more effective than passive control conditions (wait lists), but not significantly more effective than active control treatments such as psychoeducation. In contrast, there is evidence that trauma-focused face-to-face CBT is more effective than active controls, such as non-directive treatments (e.g., Ehlers et al., 2014; Schnurr et al., 2007). The average dropout rate for Internet treatments for PTSD was 22% (Kuester et al., 2016), which is comparable to the average dropout rate of 18% reported for face-to-face interventions for PTSD (Imel, Laska, Jakupcak, & Simpson, 2013).

Studies have found that therapist-supported Internet-based treatments for conditions comorbid with PTSD, such as depression, are more effective than stand-alone treatments (e.g., Gellatly et al., 2007; Johansson & Andersson, 2012). Kuester et al.'s (2016) meta-analysis found that, when compared with passive control conditions, Internet-based treatments for PTSD that offered therapist support were associated with a large effect size (*g*=0.80), compared with moderate effect sizes for stand-alone Internet-based treatments for PTSD (*g*=0.54). However, this difference was not significant, possibly due to the small number of studies. Thus, current versions of therapist-guided Internet-based treatments for PTSD have shown promising results, but have not yet been shown to have specific effects.

Trauma-focused cognitive therapy for PTSD (CT-PTSD) has been demonstrated to be highly effective and very acceptable to patients (as indicated by low dropout rates) in seven randomised controlled trials (Duffy, Gillespie, & Clark, 2007; Ehlers, Clark, Hackmann, McManus, & Fennell, 2005; Ehlers et al., 2003, 2014, 2016; Meiser-Stedman et al., 2016; Smith et al., 2007) and in routine clinical care (Ehlers et al., 2013). The treatment is typically delivered over 12 weeks in weekly sessions of 90 min with a total therapist contact time of about 18 h. An intensive version of this treatment, offered over 5–7 days, has also been shown to be feasible and effective (Ehlers et al., 2014). We have also recently shown that the therapist time required to deliver the treatment can be reduced by about 50% through self-study modules that patients complete between sessions (Ehlers et al., 2016; Wild & Ehlers, 2010).

CT-PTSD is based on Ehlers and Clark's (2000) cognitive model of PTSD, which suggests that people with PTSD perceive a current threat that has two sources, excessively negative appraisals of the trauma and/or its sequelae and particular characteristics of trauma memories that lead to re-experiencing symptoms. Cognitive strategies and behaviours (such as thought suppression, rumination, hypervigilance for threat, safety-seeking behaviours and avoidance) that are intended to reduce the sense of current threat maintain the problem by preventing change in the appraisals or trauma memory, and/or by increasing symptoms. CT-PTSD targets the three factors specified in the model. Key treatment procedures include the following:

1. An individualised case formulation based on the treatment model.
2. *Reclaiming your life assignments* that involve reclaiming or rebuilding activities and social contacts.
3. *Updating trauma memories*, a three-step procedure that includes (i) accessing memories of the worst moments during the trauma and their currently threatening meanings, (ii) identifying information that updates these meanings (information either from the course of events during the trauma or from cognitive restructuring and testing of predictions) and (iii) linking the new meanings to the worst moments in the memory. It also includes a visit to the site of the trauma.
4. *Discrimination training with triggers of re-experiencing* involves systematically spotting idiosyncratic triggers (often subtle sensory cues) and learning to discriminate between NOW (cues in a new safe context) and THEN (cue in the traumatic situation).
5. *Reducing unhelpful strategies* such as rumination, hypervigilance for threat, thought suppression and excessive precautions (safety behaviours) with behavioural experiments.

This study reports the development and piloting of a version of CT-PTSD that implements all its key procedures via the Internet, with remote support from a therapist. The Internet-delivered cognitive therapy for PTSD (iCT-PTSD) programme builds on our self-study-assisted version of CT-PTSD (for further details, see Wild & Ehlers, 2010) and our Internet-delivered version of CT for social anxiety disorder (Clark et al., 2016; Stott et al., 2013).

## Method

### Participants

A development cohort of 10 patients meeting DSM-IV (APA, 2000) criteria for PTSD worked through the Internet programme. They were recruited via two National Health Service clinics for patients with anxiety disorders or depression. Patients were assessed with the Structured Clinical Interview for DSM-IV Axis I (SCID-I; First, Spitzer, Gibbon, & Williams, 1995), with additional questions included to assess DSM-5 criteria for a diagnosis of PTSD, and Axis II disorders (SCID-II; First, Gibbon, Spitzer, Williams, & Benjamin, 1997) by three clinical psychologists with extensive experience in administering the SCID. Thirteen patients with PTSD were assessed for possible inclusion in the development cohort and three patients were excluded. Exclusion criteria (with percentage of screened patients meeting criteria in parentheses) included diagnosis of acute psychosis (0%), comorbid bipolar disorder (0%), substance dependence (0%), acute suicidality (0%), severe physical injury preventing the use of computers (*n*=1, 8%), expressed wish for face-to-face treatment (*n*=1, 8%) and PTSD diagnosed as the secondary rather than primary diagnosis (*n*=1, 8%). All participants also met DSM-5 criteria for PTSD and the caseness criteria for PTSD set out for the English *Improving Access to Psychological Therapies (IAPT*) outpatient services, as defined by a score of 33 and above on the Impact of Events Scale-Revised (IES-R; Weiss & Marmar, 1996), and eight (80%) met the caseness criteria for depression, as defined by a score of 10 or above on the 9-item Patient Health Questionnaire (PHQ-9; Kroenke, Spitzer, & Williams, 2001).

### Measures

Outcome measures were taken before and after therapy.

#### PTSD symptoms

The primary outcome measures were the PTSD Checklist for DSM-5 (PCL-5; Weathers et al., 2013) and the PTSD Symptom Scale-Interview (PSS-I; Foa, Riggs, Dancu, & Rothbaum, 1993), a structured clinical interview with good reliability and validity to assess DSM-IV criteria for PTSD (Foa et al., 1993). In addition, we report the results for the IES-R (Weiss & Marmar, 1996), as this is the PTSD measure used in IAPT services from which the sample was drawn.

#### Criteria for treatment response and remission

Remission was defined as the loss of a PTSD diagnosis on the PSS-I. Bovin et al. (2015) reported a retest reliability of *r*=0.84 for the PCL-5, which suggests a reliable change criterion of 12.38 for the SD observed in this sample. Data for the PCL-4 suggest that a 10–20-point change represents clinically significant change. For comparison with UK national data across all *Improving Access to Psychological Therapies (IAPT)* services in England, we also coded IAPT recovery criteria, which require a score of less than 33 on the Impact of Events Scale-Revised (IES-R; Weiss & Marmar, 1996) and a score of less than 10 on the PHQ-9 (see Department of Health, 2011).

#### PTSD process measures

A 20-item short version of the Posttraumatic Cognitions Inventory (PTCI; Foa, Ehlers, Clark, Tolin, & Orsillo, 1999; Kleim et al., 2013) was administered to assess negative appraisals linked to PTSD. The Response to Intrusions Questionnaire (RIQ; Clohessy & Ehlers, 1999; Murray, Ehlers, & Mayou, 2002) assesses maladaptive responses to intrusive memories (rumination, suppression and numbing).

#### General mood

The PHQ-9 (Kroenke et al., 2001) and the 7-item Generalised Anxiety Disorder questionnaire (GAD-7; Spitzer, Kroenke, Williams, & Löwe, 2006) were administered to assess depression and general anxiety.

#### Functional outcome

The Work and Social Adjustment Scale (WSAS; Mundt, Marks, Shear, & Greist, 2002) was administered pre- and post-treatment to assess functional impairment. The WSAS is a 5-item, simple, reliable and valid measure of impairment in work, family and social functioning.

#### Post-treatment exit interview

At the end of the treatment, patients were interviewed by an independent assessor about their experience of the iCT-PTSD programme, what they found helpful or unhelpful and suggestions for future improvements.

## Internet programme description

### Overview

In developing our Internet treatment programme, we had a number of objectives in mind: (1) to utilise the latest technology to implement all of the key features of CT-PTSD; (2) to substantially reduce therapist contact time yet provide an ongoing experience of therapist support; (3) to develop a user-friendly, engaging programme that could sustain the attention of patients with concentration problems, a common symptom of PTSD; (4) to facilitate patients’ engagement with trauma memory work and commonly avoided thoughts and feelings without the presence of a therapist; (5) to develop a secure, confidential programme; (6) to create a programme that could be accessed on a range of platforms and mobile devices at any time of day and any day of the week to fit with patients’ schedules; and (7) to create a user-friendly web programme for therapists, facilitating access to their patients’ completed modules and scored questionnaires while also providing a user-friendly environment for them to upload individualised trauma triggers and comment on patients’ trauma stories, hotspots and completed modules as necessary.

We adapted the modules of our self-study-assisted version of CT-PTSD (Ehlers et al., 2016; Wild & Ehlers, 2010) for use on the Internet and developed additional modules, making use of state-of-the-art technology so that the programme could implement all treatment components of CT-PTSD without relying on the presence of a therapist for a particular procedure and without compromising treatment integrity. The result is a comprehensive therapist-assisted web-based programme that engages and supports patients while significantly reducing therapist contact time. A short video illustration of the Internet programme can be viewed at http:/oxcadat.org/ptsd.

### Modules

The programme is delivered in a series of modules. The therapist usually releases one or two modules at a time for the patient to work on. The order of modules is individualised and depends on the evolving individual case formulation. Modules typically comprise educational text, patient testimonies, video illustrations, case examples, questions followed by input boxes for patients to record their answers, various types of monitoring sheets, suggestions for behavioural experiments and other assignments. Patients complete the modules, linked activities and therapist's suggestions in their own time. Thirteen core modules are designed to be completed by all patients and cover five components of treatment:

1. Introducing the treatment, setting treatment goals and normalising PTSD symptoms (two modules: *Introducing the treatment, It is all understandable*)
2. Planning activities to reclaim quality of life (one module: *Reclaiming your life*)
3. Working on the trauma memory (nine modules: *Updating memories*, part 1: telling the story of your trauma, part 2: finding your hotspots, part 3: how to update your hotspots and part 4: updating your hotspots; *Spotting memory triggers*; *Beating memory triggers*, part 1: then vs. now, part 2: then vs. now practice using the My Triggers page and part 3: tackling triggers in everyday life; *My site visit*)
4. Behavioural experiments and overgeneralised danger (two modules: *Understanding and dealing with risk*, part 1 and part 2)
5. Relapse prevention (one module: *My therapy blueprint*)

A range of optional modules focus on different cognitive themes and common comorbid problems. Four modules address difficult appraisals that require more detailed cognitive restructuring (with percentages of patients for whom they were assigned): dealing with guilt (40%), dealing with anger (10%), overcoming shame and humiliation (10%) and dealing with physical changes (0%). Three modules address common maintaining strategies: rumination (50%), dealing with alcohol (0%) and dealing with cannabis (0%). Eight modules address common comorbid problems: chronic pain and PTSD (0%), self-esteem (50%), loss (10%), overcoming sleep problems (30%), overcoming depression (20%), overcoming panic attacks (0%), dissociation (10%) and working on early memories (0%). There are up to three follow-up modules (preparing for my first, second and third follow-up) all with similar content, aimed to help the patient master difficulties in follow-up using core learning from the treatment. All modules include suggestions to take a break at predetermined points, which usually follow the completion of a discrete piece of therapeutic work (e.g., patients are encouraged to take a break after telling their trauma story rather than mid-way through).

### Special features

The programme has a number of special features to enable all procedures of CT-PTSD to be implemented. There are three special pages with unique digital options to facilitate working on the trauma memory, its worst moments and trauma triggers:

#### My Story page

The *My Story* page allows patients to write an account of their trauma, which they can edit at any time or start afresh, if they wish. The functionality allows patients to write important parts of their story in a different colour. For example, patients may choose to write the worst moments of the trauma in one colour and once they have discovered new information that changes the current meaning of their worst moments (updating information), they can go back to their story and include this information in a different colour. The therapist can also add supportive comments, questions or suggestions within the patient's text. Alternatively, patients can choose to audio record their story. They can also use the dictation function on their smartphones to record their story and upload the text to the My Story page. The My Story page includes an easily accessible audiofile with instructions to reduce the likelihood of dissociating while working on the story, reminding patients of the here and now, that they are safe and that their trauma is in the past. Patients can also upload their own reminders, such as photos or brief written messages, to remind them that they are safe.

#### My Thoughts/Hotspots page

The programme has a dedicated *My Thoughts/Hotspots* page, which allows patients to record their worst moments and work on updating their meanings. Patients can upload media (sound files and images) and write down information to provide updating information. The page captures patients’ ratings of different emotions linked to their hotspots every time they add or work on their hotspots, and the functionality allows them to create an audiofile of their whole hotspot or just of their updating information.

#### My Triggers page

The patient and therapist can upload trauma triggers in the form of images, videos or sound files to the *My Triggers* page where each trigger and linked ratings of “nowness” and distress are saved. A patient can access their individual trigger at any time and practice stimulus discrimination. Every time the patient practices stimulus discrimination with a trigger, the page captures ratings of “nowness” and distress. An easily accessible audiofile includes audio content to reduce the likelihood of dissociation and becomes available when patients work on their triggers. Patients can also upload still images to remind themselves of the here and now and that they are safe to the top of the My Triggers page, which become the first images they see when they open the page.

#### Behavioural experiments

There is a dedicated log, available at all times, for patients to plan and record behavioural experiments. Behavioural experiments are a core part of a module on dealing with the sense of heightened risk after trauma (*Understanding and dealing with risk*, part 2). Patients are encouraged to carry out experiments to update their fearful thoughts about danger. They are encouraged to test their predictions about what will happen if they drop their hypervigilance and particular safety behaviours, such as checking whether doors and windows are locked, or if they try an activity they have been avoiding, such as sitting in a car after a car accident. The module outlines five steps for planning and carrying out behavioural experiments. The primary rationale is that the patient's memory of their trauma drives thoughts, feelings and attention to danger and that it is necessary to reduce safety behaviours and avoidance to experience the actual rather than overgeneralised risk.

The five steps encourage the patient to identify a situation where they would use safety behaviours or would avoid, then make predictions about what would happen if they drop their safety behaviours or try the avoided activity or situation, test the predictions while dropping safety behaviours, note the outcome of the experiment and finally, make a note of any further experiments to plan to promote further learning.

In addition to helping patients plan experiments to test predictions about risk, therapists may also guide patients to plan experiments to test predictions about how reclaiming life activities may make them feel. For example, some patients may believe that trying 10 min of activity will make them feel more fatigued. They may then plan an experiment to test the prediction that 10 min of activity is unhelpful. Patients log experiments about risk and reclaiming life activities and the outcomes in the behavioural experiments log.

#### Progress charts

Patients complete weekly questionnaires to track symptoms, functioning and process measures online (PCL-5, IES-R, sPTCI, RIQ, GAD-7, PHQ-9, WSAS as described above). The programme automatically scores and plots a graph for each questionnaire so that patients and therapist can monitor the patient's progress.

#### Resource libraries

The site has a personal library unique to each patient and a general library of resources for all patients. The personal library holds images, audiofiles, videos and written materials, such as surveys for normalising symptoms or updating hotspots, that the patient or therapist may have uploaded during the course of treatment. The general library holds all the videos that appear in the modules and the instructional videos linked to the special pages. The general library also holds other useful resources, such as where to get help in a crisis and monitoring sheets, such as a self-esteem log, used in some of the modules.

#### Videos

Engaging and empathic video presentations and whiteboard graphical illustrations explain the central ideas and key procedures of the treatment. Video clips of therapists aim to educate the patient about a particular technique, normalise symptoms and enhance the patient's experience of therapist empathy. Whiteboard videos also aim to normalise symptoms, explain maintenance cycles, illustrate how to carry out particular therapeutic procedures, such as stimulus discrimination and visiting the trauma site, and help engage patients in emotionally challenging trauma-focused therapeutic procedures. Within each module, patients also have access to videos of previous patients, who give encouraging testimony of their experience with a particular therapeutic procedure, such as working on their trauma memory or visiting the trauma site. Further videos illustrate how previous patients (played by actors) conducted core procedures. A video is also available for people, such as friends or family, who may be supporting the patient with revisiting the trauma site. However, there is no specific requirement for support from anyone other than the remote support of the therapist.

### Therapist support

Remote therapists can view all pages on the Internet programme as if viewing them from a patient's perspective. They can then provide advice, direction and support via a range of media, including secure messaging, SMS texts, brief weekly calls and an in-built video link. The therapists are able to see when patients have logged onto the programme, what pages they have viewed, what modules they have worked on and what they did in each login. There is also a running record of behavioural experiments and other assignments planned and executed with time stamps. Each option for therapist communication has particular indications. The vast majority of therapist patient communications are asynchronous (not live at the same time). In this pilot series, therapists scheduled one weekly telephone call with patients and typically used SMS messages to send supportive comments about carrying out assignments and behavioural experiments. Emails were used to send summaries of key learning points following the weekly telephone call and a summary of next steps, such as which modules to complete next.

### Data analysis

Pre- to post-treatment changes on the outcome measures were evaluated with paired *t*-tests. For comparisons with meta-analyses, treatment effect sizes for changes in symptom and process measure scores were calculated using Cohen's *d* statistic with *d*=0.20 representing a small effect, *d*=0.50 representing a medium effect and *d*=0.80 representing a large effect (Cohen, 1988). Meta-analyses differ in whether they calculate these effect sizes as *d*=*M*_initial_ − *M*_post_/SD_pooled_, with ${\text{SD}}_{\text{pooled}}=\text{SQRT}\left(\left({\text{SD}}_{\text{initial}}^{2}+{\text{SD}}_{\text{post}}^{2}\right)/2\right)$ (e.g., Van Etten & Taylor, 1998), or, as *d*=*M*_initial_ − *M*_post_/SD_initial_ (Feske & Chambless, 1995) and we therefore report both indices.

## Results

### Characteristics of patients

Patients’ mean age was 30.4 years (SD=9.2), and the mean duration of PTSD in months was 25.5 (SD=31.4). The index trauma patients currently sought treatment for were as follows: seven patients (70%) had experienced road traffic accidents, one patient (10%) experienced the traumatic birth of her baby, one patient (10%) suffered a physical assault and one patient (10%) experienced a traumatic bereavement (murder) of a parent. Five patients (50%) reported a history of other traumas. Four patients (40%) were employed part-time, one patient (10%) was employed full-time, three patients (30%) were unemployed, one patient (10%) was on sick leave and one patient (10%) was a student. Comorbid Axis I disorders were panic disorder (30%), agoraphobia (20%), major depressive disorder (60%), generalised anxiety disorder (30%), social anxiety disorder (30%) and adult attention deficit and hyperactivity disorder (10%). Axis II disorders were obsessive–compulsive personality disorder (30%), paranoid personality disorder (10%) and avoidant personality disorder (10%). One patient (10%) had previously received brief counselling for PTSD via the telephone, and three patients (30%) had received previous treatment for other problems (psychodynamic therapy for depression, counselling for panic attacks and emotion-freedom technique for general issues). Four patients (40%) were on anti-depressant medication and remained on a stable dose throughout the treatment.

### Use of the Internet programme

Patients spent a mean of 21.7 h (SD=15.9) over an average period of 9.6 weeks (SD=2.7) working on the programme. They worked on their trauma story a mean of 5.3 times (SD=6.5) and updated a mean of 2.5 hotspots (SD=2.32). Six patients (60%) went back to the site of their trauma in person or virtually with the use of Google Street View. Patients uploaded on average 6.6 triggers (SD=6.8) and recorded on average 4.2 behavioural experiments (SD=7.2).

### Therapist activity

Therapists made a mean of 10.5 telephone calls (SD=3.9) during the course of treatment, which equated to a mean total telephone contact time of 3.2 h (191.8 min, SD=88.6). Therapists sent a mean of 20.7 (SD=11.2) emails to their patients and a mean of eight mobile SMS (SD=10.3). Their indirect contact time, which was used to review patients’ completed modules, upload triggers and to write SMS and emails, was an additional 57.4 min (SD=25.8) per patient. The indirect time is comparable to time for preparation of face-to-face sessions (e.g., reviewing notes and questionnaires) and messages. Thus, the total number of minutes therapists spent supporting patients in the course of the treatment was 4.1 h (247.5 min; SD=101.58).

### Clinical outcome

Table 1 shows the means and standard deviations for the outcome and process measures pre- and post-treatment and the *t*-values for repeated measures analyses and effect sizes of the pre- to post-treatment change.

**Table 1:** Outcome and process measures at pre- and post-treatment

	Pre-treatment		Post-treatment			Cohen's *d*^a^ (pooled SD)	Cohen's *d*^b^ (pre-treat SD)
					
Measure	*M*	SD	*M*	SD	*t* (*df*=9)		
Posttraumatic stress symptom measures							
PCL-5	47.90	11.17	15.80	15.98	5.74***	2.33	2.87
IES-R	50.05	11.37	13.19	16.03	8.40***	2.65	3.24
PSS-I	31.70	8.04	12.44	8.56	5.42***	2.32	2.40
Posttraumatic stress process measures							
sPTCI	66.00	18.38	42.90	27.24	3.81**	0.99	1.26
RIQ	24.40	5.25	7.80	7.17	7.77***	2.64	3.16
General mood measures							
PHQ-9	12.80	5.81	5.50	5.52	3.43**	1.29	1.26
GAD-7	11.60	5.56	4.40	5.17	3.71**	1.34	1.29
Work and social adjustment							
WSAS	20.50	8.72	10.58	10.12	2.57*	1.05	1.14

*** *p*<0.001,

** *p*<0.01,

* *p*<0.05.

Notes: Repeated measures *t*-tests were conducted on all measures from pre- to post-treatment (*N*=10). PCL-5=PTSD Checklist for DSM-5 (PCL-5; Weathers et al., 2013); IES-R=Impact of Events Scale-Revised (Weiss & Marmar, 1996); sPTCI=short version of Posttraumatic Cognitions Inventory (PTCI; Foa et al., 1999; Kleim et al., 2013); RIQ=Response to Intrusions Questionnaire (Foa, Ehlers, et al. 1999); PHQ-9=Patient Health Questionnaire (Kroenke et al., 2001); GAD-7=Generalised Anxiety Disorder Screener (Spitzer et al., 2006); WSAS=Work and Social Adjustment Scale (WSAS; Mundt et al., 2002).

a Cohen's *d*=*M*_initial_ − *M*_post_/SD_pooled_, with ${\text{SD}}_{\text{pooled}}=\text{SQRT}\left(\left({\text{SD}}_{\text{initial}}^{2}+{\text{SD}}_{\text{post}}^{2}\right)/2\right)$;

b Cohen's *d*=M_initial_ − *M*_post_/SD_initial_ (Feske & Chambless, 1995).

#### PTSD symptoms: clinically significant change and remission

**Fig. 1:**
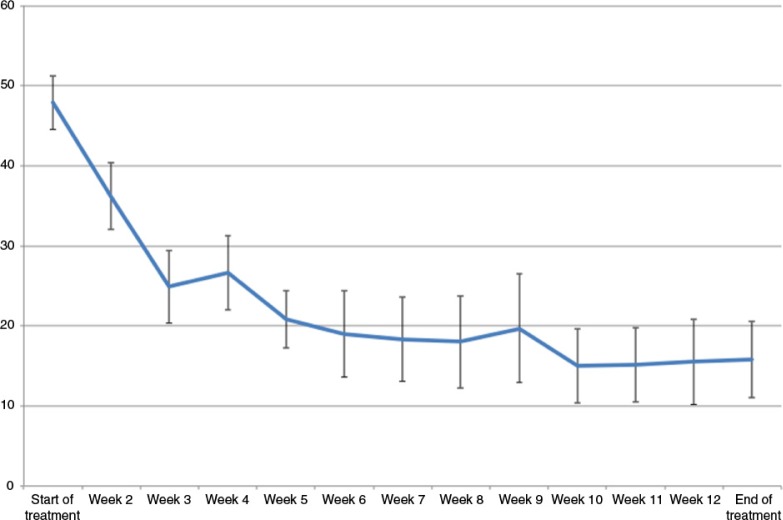
Patients’ mean PCL-5 scores over time with SEM error bars.

All PTSD measures showed very large improvement with treatment. Nearly all patients (90%) showed reliable change on the PCL-5, achieving a mean drop of 32.10 points (SD=17.67). Eight patients (80%) showed a drop of 20 points or more, satisfying criteria for clinically significant change. Repeated measures *t*-test showed a significant change on this measure, *t*(9)=5.74, *p*<0.001. Figure 1 plots the mean weekly changes on the PCL-5. At the end of the treatment, eight patients (80%) were assessed as not having PTSD by an independent assessor on the PSS-I. The same eight patients met IAPT recovery criteria. An exact McNemar's test determined that there was a statistically significant difference in the proportion of PTSD diagnoses pre- and post-intervention with significantly fewer positive PTSD diagnoses post-intervention, *p=*0.008.

#### Process measures

Patients showed significant reductions in posttraumatic appraisals and maladaptive responses to intrusive memories (Table 1) by the end of the treatment with large effect sizes. Repeated measures *t*-tests showed significant change on the PTCI, *t*(9)=3.81, *p*<0.01 and the RIQ, *t*(9)=7.77, *p*<0.001.

#### General mood

Patients made significant improvements in mood and anxiety over the course of treatment (Table 1), with large effect sizes. Repeated measures *t*-tests showed a significant change on the PHQ-9, *t*(9)=3.43, *p*<0.01 and the GAD-7, *t*(9)=3.71, *p*<0.01.

#### Functional outcome

Patients made significant gains in their work and social adjustment, with a large effect size (see Table 1). Three patients (30%) changed their employment status during the course of treatment: one patient (10%) moved from unemployed to full-time status, one patient (10%) moved from part-time to full-time work and one patient moved from sick leave to self-employed. However, overall the change in employment status from pre- to post-intervention was not significant.

#### Exit interviews

Patients reported that they liked the structure of the treatment, the different functions available on the website, the modules, the videos, planning experiments and regular contact with their therapist. One patient commented that the module on shame was particularly helpful; another patient identified the guilt and memory modules as most helpful, saying that: “The module on guilt I found very useful. Just the way it broke it all down. It showed you examples of people and things that they felt guilty about, then it asked you what you felt guilty about. It just really broke it down. And I think initially, as well, telling the story of the accident, and going back and adding all the things [updating information] was helpful. And the experiments were very helpful. And basically all of it. I can't think of anything that was unhelpful.” Patients particularly liked the therapist recording on the My Triggers page reminding them that they are safe and that their trauma is in the past with one patient saying that it helped them to stay focused when they practiced stimulus discrimination with triggers. One patient with severe concentration problems linked to ADHD reported that the take-a-break suggestions within the modules helped to pace his attention while working on the site. All patients reported that seeing video testimony of previous users was motivating and helpful, particularly when they spoke about visiting the trauma site and working on their trauma memory. There was only one suggestion for improvement, relating to a technical issue (automatic logout), which has now been resolved.

## Discussion

Our iCT-PTSD implements all the core procedures of standard CT-PTSD and was associated with significant changes on PTSD outcome and process measures. Encouragingly, the percentage of patients (80%) achieving remission from PTSD as assessed by an independent assessor and clinically significant change on a standard self-report measure of PTSD is consistent with published trials of face-to-face CT-PTSD (e.g., Ehlers et al., 2014). The majority of patients (70%) achieved significant change after 4 weeks of treatment, which may indicate a more immediate treatment response than is observed in face-to-face CT-PTSD and may relate to the frequency with which patients worked on the programme. Patients also achieved significant change on process measures, in line with the hypothesis that the treatment reverses the core maintaining factors specified in Ehlers and Clark's (2000) cognitive model of PTSD. There was also a substantial improvement in social and occupational functioning overall, and 30% of patients benefited from a positive change in employment status by the end of the treatment. However, it should be noted that it is not possible to conclude whether or not the changes in occupational status were attributable to the treatment effect alone. The treatment was acceptable to patients and there were no dropouts, a promising outcome given that existing Internet treatments for PTSD show an average dropout rate of 22% (Kuester et al., 2016). Our pilot cohort required 3.2 h of direct therapist contact via phone calls, that is, about 20% of the face-to-face treatment time in standard CT-PTSD (e.g., Ehlers et al., 2014). In addition, therapists spent about an hour reviewing each patient's modules and sending messages, which is comparable to preparation time for sessions and message writing for the standard course of face-to-face sessions. While the amount of time patients spent on their treatment was greater than the amount of time therapists spent supporting them, it appears to be comparable to the amount of time patients may spend in a standard course of CT-PTSD (e.g., Ehlers et al., 2014).

Creating a programme that could safely engage patients with probable concentration problems linked to their PTSD symptoms without the presence of a therapist was a significant challenge. We were keen to create a trusting therapist relationship with less therapist time than standard face-to-face treatment. To facilitate these requirements, we drew on available web-based functionality, created engaging, illustrative videos and provided regular, brief therapist contact via email, telephone and mobile SMS. It is our impression that this three-pronged approach was necessary to meet our development aims. We paid particular attention to the faithful implementation of three trauma-focused procedures: recounting and updating the trauma memory, discrimination training with trauma triggers and visiting the site of the trauma. Therapists adapted iCT-PTSD to patients’ individual case formulations, and consistent with CT-PTSD, a significant part of the treatment focused on particular cognitive appraisals relevant to the individual patient and maintaining behaviours. Comorbid problems were addressed as needed. The most frequently released optional modules were overcoming rumination, dealing with guilt and self-esteem.

Treatments of PTSD vary widely in the degree of exposure to trauma memories. Some non-trauma-focused interventions have shown therapeutic effects (for a meta-analysis see Bisson, Roberts, Andrew, Cooper, & Lewis, 2013), but overall trauma-focused PTSD treatments, which include some exposure to trauma memories and triggers, have the best evidence of efficacy and have been shown to be more efficacious than non-directive treatments (Bisson et al., 2007, 2013; Ehlers et al., 2010). Like standard CT-PTSD, iCT-PTSD includes some exposure, but much less than in prolonged exposure (Foa, Ehlers, et al., 1999) and other exposure therapies (e.g., Marks, Lovell, Noshirvani, Livanou, & Thrasher, 1998), and the exposure is closely interwoven with cognitive work and the patient's attention is directed differently. The focus is on updating the meanings of the worst moments of the trauma and on discriminating triggers and cues present during the trauma, rather than on repeated exposures.

Therapists provided regular and brief contact with patients via a weekly telephone call at a prearranged time. The total time of 4.1 h therapists spent supporting patients was similar to the mean time therapists spent supporting patients in iCT-SAD (Stott et al., 2013), consistent with early trials of Internet-based treatments for PTSD (e.g., Klein et al., 2010), and substantially less than the therapist time of 18 h of face-to-face sessions plus preparation time in a course of standard CT-PTSD (e.g., Ehlers et al., 2014).

Consistent with the findings of Knaevelsrud and Maercker (2007), patients reported feeling well supported by their therapists even though the majority of communication was asynchronous (not live at the same time) and contact time with clients was substantially less than in face-to-face treatment. Asynchronous communication has numerous advantages over live interaction. Therapists could log on to the site to review a patient's progress and send brief messages of encouragement whenever they wished (Stott et al., 2013). Patients were also free to contact therapists by secure messages within the programme whenever was convenient for them to do so. Asynchronous communication likely facilitates patients working on the programme regularly since therapists are able to respond briefly and often in writing rather than less frequently and orally as occurs with live conversation. Patients can refer back to their written messages of support and guidance as they work through the programme, which may encourage continuous engagement rather than once weekly logins. Importantly, the regular, brief contact afforded by asynchronous therapist patient communication may help reduce dropouts. However, randomised controlled trials are needed to evaluate further the efficacy of and rates of dropout linked to a course of iCT-PTSD.

Live conversations have disadvantages in that it can be time consuming to co-ordinate patient and therapist schedules and if a scheduled call is missed, it can be difficult for the therapist to rearrange an alternative call in the same day or week, which may delay patients’ work on the programme. Asynchronous therapist–patient communication gives therapists greater flexibility in how they manage their time. This together with the overall reduced therapist contact time seen in iCT-PTSD in this pilot series suggests the possibility that therapists could treat more patients with iCT-PTSD than standard CT-PTSD. For every patient seen with face-to-face CT-PTSD, up to five patients could be seen with iCT-PTSD. However, this may be an over-simplistic calculation as Internet treatment involves a change in the way that therapists deploy their time, as well as an overall reduction in the total amount of time per patient (Stott et al., 2013).

It is also interesting to note that most patients in this pilot series responded quickly to treatment. The majority of patients had achieved clinically significant change after 4 weeks of treatment. It is too early to conclude whether or not iCT-PTSD leads to faster recovery from PTSD than CT-PTSD since the treatment needs to be evaluated with a larger sample over a longer period of time and in a randomised controlled trial. However, it is plausible that iCT-PTSD could help to facilitate treatment response over a shorter time period than standard CT-PTSD since patients are able to work on their treatment as often as they wish with regular brief remote guidance from their therapist. This frequency of contact is not possible with face-to-face CT-PTSD.

Internet-delivered treatment may also have the advantage that it ensures treatment fidelity, as the content of the modules is the same for all patients. The 80% recovery rate according to IAPT criteria compares favourably with the 38% recorded for PTSD patients across the IAPT services in England (Health and Social Care Information Centre, 2015). While we cannot rule out that therapist experience and selection criteria such as being able to read and write in English may have contributed to this difference, ensuring that all patients received essential interventions targeting the factors maintaining their PTSD may have played a role in achieving the positive results.

Of course, the conclusions that can be drawn from this study are limited since the pilot cohort was small and there was no control condition. It thus cannot be determined whether the treatment was causally responsible for patients’ improvements. Follow-up assessments and randomised controlled evaluations are needed to assess the value of iCT-PTSD and the stability of treatment effects. It is unknown, which patients would benefit most from iCT-PTSD, and future research will need to investigate possible predictors of poorer treatment response. The treatment requires the patient to read and write and some patients may find this challenging (Wild & Ehlers, 2010). It is also possible that patients with multiple traumas or multiple comorbid disorders may be slower to respond with this new treatment because problems that may affect concentration may also interfere with the ability to carry out challenging procedures without the presence of a therapist. However, patients in this cohort had comorbid conditions and multiple traumas and responded well to the treatment. Access to modules to support the work on comorbid problems, such as modules on depression or loss, may have been beneficial for patients with comorbid problems.
